# Comparing the Effectiveness and Safety of Different Third-Line Management Options for Patients with Metastatic Colorectal Cancer: A Systematic Review and Reconstructed Individual Patient Data Meta-Analysis

**DOI:** 10.3390/cancers18142204

**Published:** 2026-07-08

**Authors:** Maen Abdelrahim, Abdullah Esmail, Nour Mustafa, Ebtesam Al-Najjar, Yazan Hamdaneh, Zaid Alabed, Hikmat Abdel-Razeq, Asem Mansour

**Affiliations:** 1 Section of GI Oncology, Department of Medicine, Houston Methodist Cancer Center, Houston, TX 77030, USA; aesmail@houstonmethodist.org; 2 Department of Medicine, Weill Cornell Medical College, New York, NY 10065, USA; 3 Faculty of Medicine, The University of Jordan, Amman 11942, Jordan; 4 Department of Internal Medicine, King Hussein Cancer Center, Amman 11941, Jordan; 5 Department of Radiology, King Hussein Cancer Center, Amman 11941, Jordan

**Keywords:** metastatic colorectal cancer, third-line therapy, trifluridine/tipiracil, fruquintinib, regorafenib, overall survival

## Abstract

Background: Metastatic colorectal cancer (mCRC) that progresses after standard first- and second-line therapies carries a poor prognosis, and multiple third-line treatment options are available without a clearly established optimal strategy. This study aimed to comprehensively compare the efficacy and safety of currently approved third-line treatments using reconstructed individual patient data (reconstructed IPD). Methods: A systematic review and reconstructed IPD meta-analysis of phase III randomized controlled trials was conducted in accordance with PRISMA-IPD guidelines. Kaplan–Meier survival curves were digitized and reconstructed using validated methodologies. The primary endpoint was overall survival (OS), while secondary endpoints included progression-free survival (PFS) and the incidence of grade ≥3 treatment-related adverse events (AEs). Results: A total of 3338 patients were included across five treatment groups: regorafenib (*n* = 650), fruquintinib (*n* = 739), trifluridine/tipiracil (TAS-102; *n* = 780), TAS-102 plus bevacizumab (*n* = 246), and pooled placebo (*n* = 923). TAS-102 plus bevacizumab demonstrated the greatest benefit (OS HR: 0.43, 95% CI: 0.35–0.50; median OS: 10.89 months; PFS HR: 0.20, median PFS: 5.51 months). Fruquintinib (OS HR: 0.67; median OS: 7.97 months; PFS HR: 0.32; median PFS: 3.70 months) and TAS-102 (OS HR: 0.70; median OS: 7.2 months; PFS HR: 0.41; median PFS: 2.09 months) showed comparable survival benefit to regorafenib (OS HR: 0.68; median OS: 7.04 months; PFS HR: 0.39; median PFS: 2.16 months), with fruquintinib demonstrating superior PFS versus regorafenib (HR: 0.71, *p* < 0.001). Grade ≥3 AEs were highest with TAS-102 plus bevacizumab (72.36%) and lowest with fruquintinib (34.02%), with hematologic toxicity predominating in TAS-102-based regimens and hypertension and hand–foot skin reactions more common with tyrosine kinase inhibitors. Conclusion: TAS-102 plus bevacizumab was associated with the greatest survival benefit in this indirect comparison and appears to be the most effective option for eligible patients. However, these findings should be interpreted cautiously given the absence of direct head-to-head trials. Among tyrosine kinase inhibitors, fruquintinib demonstrated superior PFS compared with regorafenib, while OS was comparable.

## Simple Summary

Patients with advanced colorectal cancer that spreads and worsens after standard initial therapies face limited treatment options and poor outcomes. While several medications are approved for subsequent treatment, doctors lack clear guidance on which choice works best because these drugs have never been compared directly in clinical trials. This study analyzed data from past large-scale clinical trials involving 3338 patients to compare the effectiveness and safety of these therapies. We found that combining a standard oral chemotherapy drug with an intravenous targeted antibody treatment provides patients with the longest survival and the best disease control. For patients who cannot take this combination, an oral targeted drug called fruquintinib offers superior disease control compared to an alternative option called regorafenib. These findings provide comparative evidence that may help inform treatment selection while highlighting the need for direct comparative trials.

## 1. Introduction

Colorectal cancer (CRC) is the third most prevalent cancer worldwide and remains the second most common cause of cancer-related death globally. According to 2025 epidemiological updates, the global burden of CRC continues to rise, particularly in younger populations, with over 2 million new cases diagnosed annually [1,2]. There are many contributing factors for the development of colon cancer, such as family history, smoking, alcohol consumption, genetic mutations, and inflammatory bowel diseases [3,4,5,6,7,8,9]. While surgical resection offers only curative potential, a significant proportion of patients present with metastatic disease at diagnosis or eventually progress despite optimal frontline therapy. Patients who progress on standard first- and second-line therapies require evidence-based third-line options to maintain quality of life and extend survival [10,11,12,13]. Despite advances in systemic therapy, the prognosis of patients with metastatic colorectal cancer (mCRC) who progress beyond first- and second-line treatment remains poor. The standard of care for mCRC includes a combination of chemotherapy regimens such as leucovorin (folinic acid), fluorouracil (5-fluorouracil), oxaliplatin (FOLFOX), or leucovorin (folinic acid), fluorouracil (5-fluorouracil), and irinotecan (FOLFIRI) [10]. The increasing number of available third-line treatment options has introduced significant complexity in clinical decision-making.

Targeted therapy, chemotherapy alone or in combination with tyrosine kinase inhibitors (TKIs), is used as a third-line treatment. Trifluridine/tipiracil (TAS-102) chemotherapy that combines trifluridine, a cytotoxic nucleic acid analog, and tipiracil, a thymidine phosphorylase inhibitor that prolongs the action of trifluridine, were used for refractory CRC [14]. The SUNLIGHT trial evaluated the benefit of adding bevacizumab, a monoclonal antibody that inhibits vascular endothelial growth factor (VEGF), to previous chemotherapy [15]. Regorafenib, a multikinase inhibitor targeting VEGFR-1, -2, and -3, and fruquintinib, a selective VEGFR inhibitor, have also been studied in randomized trials against placebo in this setting [14,15,16,17,18].

Although several systematic reviews and network meta-analyses (NMAs) have evaluated third-line therapies for mCRC, the optimal treatment strategy remains uncertain. Guidelines from the National Comprehensive Cancer Network (NCCN) and the European Society for Medical Oncology (ESMO) recognize these agents as viable options; however, they offer limited guidance on treatment preference or sequencing due to the absence of direct head-to-head comparisons. Existing analyses are further constrained by study heterogeneity, reliance on aggregate-level data, and the exclusion of recently published phase III trials. Reconstructed individual patient data (IPD) meta-analysis offers a methodologically rigorous approach that overcomes several of these limitations by enabling granular survival estimation and more precise indirect treatment comparisons at the patient level. Therefore, this study aimed to comprehensively compare the efficacy and safety of currently approved third-line treatment options for patients with mCRC, including regorafenib, fruquintinib, TAS-102 monotherapy, and TAS-102 plus bevacizumab, through a systematic review and reconstructed IPD meta-analysis of phase III randomized controlled trials, with the goal of providing an indirect comparative assessment of their efficacy and safety to help inform treatment selection in this challenging patient population.

## 2. Methodology

### 2.1. Study Design and Search Strategy

Reconstructed individual data pooled analysis of phase III clinical trials was performed on selected studies following the Preferred Reporting Items for Systematic Reviews and Meta-Analyses (PRISMA) and reporting guidelines for reconstructed IPD (PRISMA-IPD). The protocol was not prospectively registered in PROSPERO or any other systematic review registry.

A systematic review was conducted on PubMed, Scopus, and Embase using the following search protocol: “((Colorectal cancer or colon cancer or CRC) AND ((Third-Line treatment) OR (3rd-line treatment) OR (Third-line management) OR (3rd-line management) OR (Third-line therapy) OR (“regorafenib,” “fruquintinib,” “TAS-102,” “trifluridine tipiracil”)))”. The last literature search was conducted in July 2025. Only studies published in English were included. Duplicate records identified across databases were removed before title and abstract screening. Studies meeting the following criteria were selected: (1) phase III randomized clinical trials; (2) adult patients who are older than 18 years; (3) patients with metastatic or unresectable colorectal cancer (uCRC) who are unresponsive to at least two lines of treatment; (4) and available Kaplan–Meier plots with number at risk tables for overall survival (OS) and progression-free survival (PFS).

### 2.2. Data Extraction and Risk of Bias

Data extraction and risk of bias assessment were independently performed by two investigators following a two-stage screening process. In the first stage, titles and abstracts were screened for eligibility, followed by full-text reviews of all potentially eligible studies. Any disagreements regarding study inclusion were resolved through consensus or adjudication by a third independent reviewer. Reasons for full-text exclusion included phase II study design, non-randomized studies, ineligible patient populations, and unavailable survival data when applicable.

The two reviewers independently extracted the following data from each included study: study identification information (journal name, publication date, country of origin, and ethics approval status); patient characteristics (sample size, age, and sex); intervention details (fruquintinib, regorafenib, trifluridine/tipiracil monotherapy, or trifluridine/tipiracil plus bevacizumab); comparator (placebo with best supportive care); and clinical outcomes (overall survival, progression-free survival, and treatment-related adverse events). For studies with multiple publications, only the most recent and complete report was used as the primary data source.

Risk of bias assessment of included studies was done using the Cochrane Risk of Bias (version 2) tool for randomized clinical trials (RCTs) Sterne JAC et al. [19].

### 2.3. Time to Event Outcomes Reconstruction

Using a method developed by Guyot et al. and implemented by Liu et al., time to event outcomes (OS and PFS) were estimated using a IPDfromKM web tool for graphical reconstruction from reported Kaplan–Meier plots for each group of each study, which was guided by previously published studies Zhao JJ et al., Nichetti F et al., Raimondi et al., and Pietrantonio et al. [20,21,22,23,24,25]. The data reconstruction was chosen and performed independently by two of our investigators, and the best reconstruction was selected. To overcome potential inaccuracies in survival reconstruction, a two-step validation approach was applied. First, the reconstructed Kaplan–Meier curves were visually compared with the published curves. Second, the pooled reconstructed hazard ratio of each study was compared with the published hazard ratios from the original trials (provided in Supplementary File S1). IPDs of the same treatment groups and placebo from different trials were pooled.

### 2.4. Data Analysis

The primary endpoint of this analysis was OS, defined as the time from treatment initiation to death from any cause or last follow-up within the study observation period for each treatment group.

Secondary endpoints included PFS, defined as the time from treatment initiation to disease progression, death, or last follow-up within the observation period, and the incidence of grade ≥3 treatment-related adverse events (AEs) for each group.

Studies that have not reported specific adverse event (AE) data were excluded from the corresponding safety analyses.

Pooled OS and PFS curves were estimated with the Kaplan–Meier method, while the reverse Kaplan–Meier estimator was used to quantify the median follow-up. All outcomes were compared using global log-rank tests, each arm’s outcome was investigated with Cox proportional hazards regression models, and individual patient’s trial data was included as a random variable to account for interstudy differences, as was performed in previous studies Nichetti et al., Pietrantonio et al., and Raimondi et al. [22,24,25]. Tukey’s pairwise test was used to conduct pairwise log-rank tests between all the different arms concurrently.

Treatment arms were pooled into four experimental groups (regorafenib monotherapy, fruquintinib monotherapy, TAS-102, and TAS-102 plus bevacizumab), while placebo arms of the included studies were pooled to form the control group.

Moreover, to show that our OS and PFS findings are significant, power and potential sample size analyses were conducted using the estimated treatment effect size from the Cox proportional hazards models of the derived subgroups. Specific drug experimental subgroups and class experimental subgroups were compared to a pooled control TKI subgroup, and the study power was evaluated using hazard ratios (HRs) of the comparison group with alpha = 0.05. Sensitivity analyses were also performed. In case of similar outcomes between two regimens (HR: 0.90–1.10), a non-inferiority design was adopted.

Rates of grade ≥3 treatment-related AEs were pooled using random-effects single-proportion meta-analysis and compared with χ^2^ tests. Statistical heterogeneity across studies was assessed using the I^2^ statistic and Cochran’s Q test.

*p* = 0.05 was set as the statistical significance threshold, and all statistical tests were two-sided. The analysis was conducted using R software version 4.4.2 (R Foundation for Statistical Computing, Vienna, Austria). A list of R packages used for the analysis is provided in Supplementary File S1.

## 3. Results

### 3.1. Study Selection and Characteristics

**Figure 1:**
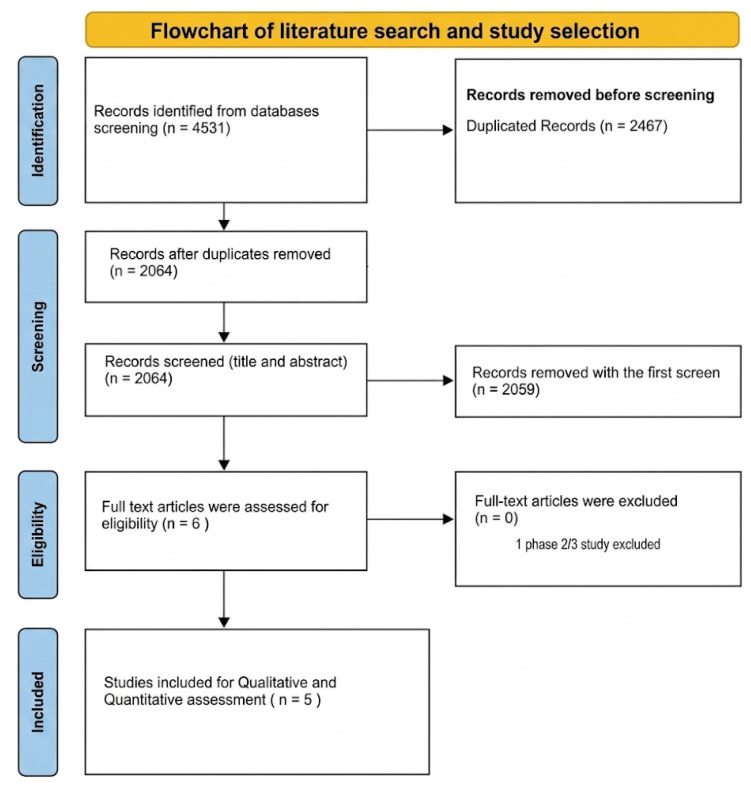
PRISMA flowchart.

The PRISMA flowchart is shown in Figure 1. Title and abstract screening were done on 4531 studies, and five studies were included in the main analysis; the characteristics of the included studies are shown in Table 1. The ReDOS trial was excluded during full-text screening due to it being a phase II trial.

A total of 3338 patients were included across all analyses: 739 patients received fruquintinib, 650 received regorafenib, 780 received TAS-102, 246 received TAS-102 plus bevacizumab, and 923 received placebo.

As shown in Supplementary Figure S1, the risk of bias analysis showed low bias in all included studies. The graphical reconstruction algorithm resulted in similar patient level data to the included trials’ OS and HRs. Moreover, a nearly complete overlap was observed between reconstructed survival curves and matched cohorts in the original plots, as shown in Supplementary Figures S2–S11.

### 3.2. Survival Outcomes

**Figure 2:**
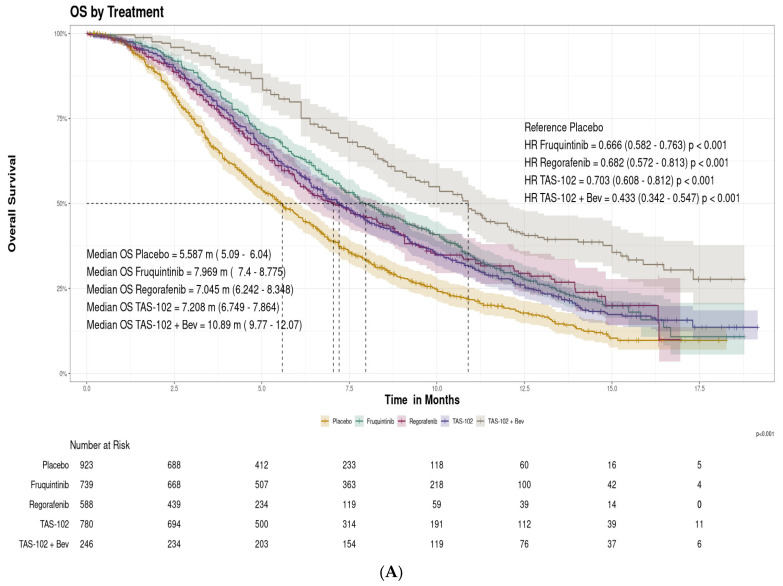

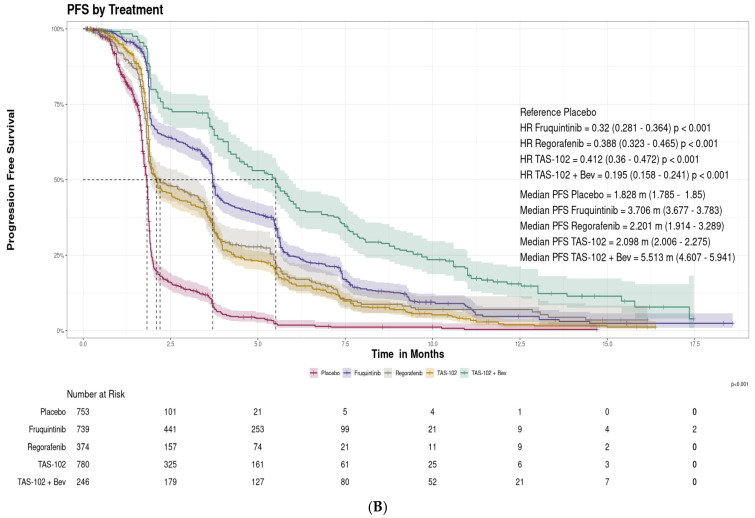
Kaplan–Meier plots for (**A**) overall survival (OS) and (**B**) progression-free survival (PFS) for patients receiving TAS-102, TAS-102 + Bev, regorafenib, fruquintinib, and placebo.

The median overall survival (mOS) for all arms was 7.18 months (95% CI: 6.84–7.40). The lowest mOS was for placebo (5.59 months; 95% CI: 5.09–6.04), while fruquintinib and regorafenib’s mOS was 7.97 months (95% CI: 7.40–8.78) and 7.05 months (95% CI: 6.24–8.35), respectively. The mOS for TAS-102 was 7.21 months (95% CI: 6.75–7.86), and TAS-102 plus bevacizumab had the highest median survival (10.89 months; 95% CI: 9.77–12.07) with a global *p*-value < 0.001 (Figure 2A).

Additionally, the median progression-free survival (mPFS) for all arms was 2.07 months (95% CI: 2.00–2.14). The lowest mPFS was for placebo (1.83 months; 95% CI: 1.79–1.85), followed by TAS-102 (2.10 months; 95% CI: 2.01–2.28), regorafenib (2.20 months; 95% CI: 1.91–3.29), and fruquintinib (3.71 months; 95% CI: 3.68–3.78), while TAS-102 plus bevacizumab had the highest mPFS (5.51 months; 95% CI: 4.61–5.94) with a global *p*-value < 0.001 (Figure 2B).

Compared to placebo, all experimental arms were significantly superior, with TAS-102 plus bevacizumab and fruquintinib having the lowest HR for OS (HR = 0.43, 0.34–0.55, *p* < 0.001; HR = 0.67, 0.58–0.76, *p* < 0.001) and PFS (HR = 0.20, 0.16–0.24, *p* < 0.001; HR = 0.32, 0.28–0.36, *p* < 0.001), respectively, as shown in Table 2.

Pairwise analysis showed that TAS-102 plus bevacizumab had significantly better OS and PFS than all of the other interventions (*p* < 0.001), while the other interventions did not show significant differences between each other in terms of OS and PFS, as shown in Supplementary Table S1.

### 3.3. Safety Analysis

Safety analysis showed TAS-102 plus bevacizumab had the highest rate of grade 3/4 AEs (72.4%) while fruquintinib had the lowest rate of grade 3/4 AEs (34%). However, fruquintinib had the highest rate of grade 3/4 hypertension (15.5%). TAS-102 plus bevacizumab also had the highest rate of grade 3/4 neutropenia (43.2%) and the second highest rate of grade 3/4 anemia (6.1%). Table 3 shows that regorafenib had the highest rate for fatigue, AST and ALT increase, and hand–foot skin reaction. Extended AE tables are provided in Supplementary File S1.

## 4. Discussion

mCRC refractory to first- and second-line therapy represents a clinically challenging setting with limited survival expectations and multiple available treatment options without a clearly established treatment hierarchy. To our knowledge, this systematic review and reconstructed IPD meta-analysis represents one of the most comprehensive and up-to-date indirect comparisons in this setting. We demonstrated that all currently approved third-line regimens, including TAS-102 monotherapy, TAS-102 plus bevacizumab, regorafenib, and fruquintinib, conferred statistically significant and clinically meaningful improvements in OS and PFS compared with placebo. Most notably, TAS-102 plus bevacizumab emerged as the most effective regimen across all survival endpoints, supporting its consideration as a promising third-line option while recognizing that these conclusions are based on indirect comparisons.

### 4.1. Overview of Subgroup Analyses

We performed four subgroup analyses that interrogated distinct clinical questions under the broader umbrella of third-line mCRC therapy options. The first analysis compared TAS-102 monotherapy, TAS-102 plus bevacizumab, TKIs as a whole class without examining each agent alone, and placebo. The second analysis specifically contrasted regorafenib, fruquintinib, and placebo, focusing on head-to-head indirect comparisons of the two approved TKIs in this setting. The third analysis performed another indirect comparison of regorafenib versus fruquintinib in the TKI cohort alone but without a placebo arm. Finally, the fourth and most expansive analysis integrated all five treatment groups—TAS-102, TAS-102 plus bevacizumab, regorafenib, fruquintinib, and placebo—to provide a unified and comprehensive assessment of all available third-line options. Together, these analyses can help clinicians to contextualize the relative advantages and side effects of each agent and combination regimen.

### 4.2. TAS-102 Monotherapy

TAS-102 is an oral nucleoside analog that exerts its antitumor effect through dual mechanisms: trifluridine is incorporated into DNA and inhibits thymidylate synthase, while tipiracil inhibits thymidine phosphorylase, preventing rapid degradation of trifluridine [26]. In the pivotal RECOURSE trial, TAS-102 demonstrated a significant OS benefit over placebo in patients with previously treated mCRC, establishing it as a standard third-line option [14]. Our findings are consistent with the RECOURSE trial and confirm that TAS-102 remains an effective third-line treatment option. Although PFS improved, the benefit was modest, supporting its role as one of several effective third-line treatment options [14].

### 4.3. TAS-102 Combined with Bevacizumab

The most important finding of this study was the superior efficacy of the TAS-102 plus bevacizumab combination compared with the other evaluated third-line regimens. This finding is consistent with the phase III SUNLIGHT trial, which established the addition of bevacizumab to TAS-102 as an effective strategy for improving survival in patients with refractory mCRC [15]. The enhanced efficacy of this combination likely reflects complementary mechanisms of action, with trifluridine disrupting DNA synthesis and bevacizumab inhibiting VEGF-mediated tumor angiogenesis, potentially improving drug delivery and maintaining a less favorable microenvironment for tumor growth [15]. However, this improved efficacy was accompanied by a higher incidence of hematologic toxicities, particularly neutropenia and anemia, emphasizing the importance of careful patient selection, close hematologic monitoring, and balancing efficacy with treatment-related toxicity. Our findings further support the role of TAS-102 plus bevacizumab as an important third-line treatment option while highlighting the need to balance its efficacy against its toxicity profile.

### 4.4. Tyrosine Kinase Inhibitors: Regorafenib and Fruquintinib

Regorafenib, a multikinase inhibitor targeting VEGFR, PDGFR, FGFR, KIT, RET, and RAF, was the first oral targeted agent approved for refractory mCRC following the CORRECT trial in 2012 [16]. Its approval in Asian populations was subsequently supported by the CONCUR trial, which demonstrated particularly favorable outcomes in that population [27]. Our findings confirm that regorafenib remains an effective third-line treatment option for patients with refractory mCRC.

Fruquintinib, a highly selective inhibitor of VEGFR-1, -2, and -3, was approved based on the phase III FRESCO trial conducted in China and subsequently validated globally through the FRESCO-2 trial [17,18]. Our findings suggest that fruquintinib provides improved disease control, particularly with respect to PFS, which may reflect its greater selectivity for VEGFR inhibition. Nevertheless, both agents demonstrated meaningful clinical benefit and remain important treatment options in the third-line setting.

### 4.5. Indirect Comparison: Regorafenib vs. Fruquintinib

A dedicated subgroup analysis indirectly compared regorafenib and fruquintinib without a placebo comparator. Although fruquintinib demonstrated superior PFS, no statistically significant OS advantage was observed between the two TKIs. This PFS benefit may reflect the greater selectivity of fruquintinib for VEGFR inhibition [17,18].

Although the ReDOS trial was excluded from the quantitative meta-analysis because it was a phase II study, its findings provide important contextual evidence that dose-escalation strategies may improve the tolerability of regorafenib without compromising efficacy. In ReDOS, dose escalation was associated with an improved safety profile, including lower rates of fatigue, hand–foot skin reaction, and hypertension, while maintaining clinical benefit, suggesting that dose optimization is an important consideration when prescribing regorafenib [28].

### 4.6. Safety and Tolerability

The safety profiles of the agents examined in this analysis are distinct and clinically important when selecting a therapy for individual patients. Hematologic toxicities, particularly neutropenia and anemia, were most pronounced in the TAS-102 plus bevacizumab arm, consistent with the myelosuppressive properties of trifluridine and the known vascular effects of bevacizumab. In the SUNLIGHT trial, grade 3 or 4 neutropenia occurred in approximately 43% of patients receiving the combination, reinforcing the need for routine complete blood count monitoring [15].

In contrast, TKI-class toxicities predominated in the regorafenib and fruquintinib arms. HFSR and hypertension were the hallmark AEs for both agents, with regorafenib exhibiting higher rates of HFSR (16.4%) and fatigue (6.1%), while fruquintinib was associated with prominent hypertension (15.5%). Fruquintinib’s narrower kinase selectivity, compared to regorafenib’s broader multi-target inhibition, may partly explain the differences in off-target toxicity, particularly the lower incidence of hepatotoxicity with fruquintinib. Diarrhea was a common toxicity across all TKI regimens but was rarely dose-limiting.

TAS-102 monotherapy was generally associated with manageable gastrointestinal toxicities (nausea, decreased appetite, and fatigue) and significant but predictable hematologic toxicities (grade 3/4 neutropenia in approximately 38% of patients in RECOURSE), with a toxicity profile that many clinicians consider more acceptable than that of regorafenib in frail or older patients [14]. These differences highlight the need for individualized treatment selection based on patient comorbidities, performance status, and prior toxicity history. The importance of balancing efficacy with treatment-related toxicity has also been emphasized across recent systematic reviews in colorectal tumor management [29].

### 4.7. Comparison with Prior Meta-Analyses

Our findings should be interpreted in the context of recent network meta-analyses evaluating third-line therapies in mCRC, including the study by Gao et al. (2023) and the more recent analysis by Hoyek et al. (2025) [30,31]. The NMA by Gao et al., which included nine phase II/III randomized trials up to May 2023, similarly identified TAS-102 plus bevacizumab as the most effective regimen [30]. These results are consistent with our findings, reinforcing the robustness of this combination as the leading third-line option. However, that analysis relied on aggregate-level data and Bayesian indirect comparisons, whereas our study utilized reconstructed IPD, allowing for more granular survival estimation and direct reconstruction of Kaplan–Meier curves across pooled cohorts.

More recently, the NMA by Hoyek et al. (2025) incorporated modern regimens including FTD-TPI ± bevacizumab, regorafenib (standard and dose-escalation), and fruquintinib, reflecting a treatment landscape closer to contemporary practice. While their study also evaluated comparative efficacy across these agents, it remained limited by trial-level comparisons and conventional NMA assumptions, without patient-level reconstruction [31]. In contrast, our analysis not only confirms the superiority of TAS-102 plus bevacizumab but also provides quantitative survival estimates and head-to-head indirect comparisons at the IPD level, including a more precise assessment of the PFS advantage of fruquintinib over regorafenib, which is less consistently quantified in prior NMAs.

Importantly, both prior analyses primarily emphasize relative ranking of treatments, whereas our study extends these findings by providing absolute survival estimates, detailed hazard ratios, and pooled safety event rates, enabling clearer clinical interpretation. Taken together, while our results are broadly consistent with these NMAs in identifying TAS-102 plus bevacizumab as the most effective regimen, our study adds incremental value through updated evidence integration, IPD-based methodology, and more clinically actionable comparisons across efficacy and toxicity domains.

### 4.8. Clinical Implications and Treatment Selection

Our findings suggest that TAS-102 plus bevacizumab may be an appropriate third-line treatment option for patients with mCRC who are fit enough to tolerate combination therapy, as it demonstrated the longest median OS (10.89 months) and PFS (5.51 months) among the evaluated regimens in this indirect comparison. However, these findings should be interpreted with caution, as direct randomized head-to-head comparisons are lacking. Although TAS-102 plus bevacizumab demonstrated the greatest survival benefit, it was also associated with the highest rate of grade 3/4 adverse events, particularly hematologic toxicity. For patients who are unable to receive bevacizumab because of contraindications, such as uncontrolled hypertension, recent arterial thromboembolism, or active bleeding, TAS-102 monotherapy or fruquintinib may represent clinically meaningful alternatives. Fruquintinib demonstrated the lowest overall grade 3/4 adverse event burden but was associated with a higher incidence of hypertension. Regorafenib also remains a reasonable treatment option, particularly when dose-optimization strategies, such as the dose-escalation approach evaluated in the ReDOS trial, are used to improve tolerability [28]. Overall, treatment selection should be individualized by balancing efficacy, toxicity profile, patient comorbidities, and treatment tolerability rather than following a fixed treatment hierarchy.

It is also important to note that the choice of third-line therapy may be influenced by the sequence of prior treatments, the mutational profile of the tumor (such as RAS/BRAF/MSI status), and regional drug availability and reimbursement considerations. Fruquintinib’s global approval through the FRESCO-2 trial has expanded access beyond Asian populations, making it a viable option for a broader international patient population [17,18].

Overall, these findings align with the broader trend toward personalized colorectal cancer care, in which treatment decisions are increasingly individualized to optimize efficacy while minimizing treatment-related toxicity and morbidity [32].

Patient-related factors, including age, frailty, comorbidities, and treatment tolerability, should also be considered when individualizing treatment decisions, consistent with recent evidence emphasizing personalized management strategies in colorectal cancer [33].

### 4.9. Strengths and Limitations

A primary strength of our study is the utilization of an IPD reconstruction tool to analyze Kaplan–Meier survival curves from the studies we included. This methodological approach allows for a more granular assessment of survival over time than standard aggregate data meta-analyses, providing a more precise estimation of the targeted endpoints. Furthermore, the inclusion of a large number of patients provides the statistical power necessary to discern subtle but clinically significant differences in safety and efficacy between the different regimens compared.

Several limitations require consideration when interpreting the findings of our study. To begin with, all cross-trial comparisons are indirect and subject to the inherent methodological limitations of such analyses, including between-trial heterogeneity in patient characteristics, prior treatment regimens, and supportive care standards. Although IPD reconstruction from Kaplan–Meier curves using validated methodology allowed for individual-level cross-study comparisons, this approach cannot fully substitute for prospective head-to-head randomized trials [20].

In addition, the included studies span a decade (2012–2023), during which treatment paradigms including the emergence of immunotherapy, the use of anti-EGFR rechallenge, and evolving criteria for third-line eligibility have changed significantly. The extent to which differences in prior therapies across trials may have influenced baseline prognosis and outcomes in each treatment arm cannot be fully quantified.

Moreover, our analysis was restricted to phase III randomized controlled trials. Although the phase II ReDOS trial was not eligible for inclusion in the pooled efficacy analyses, its findings are discussed qualitatively because they provide clinically relevant information regarding regorafenib dose optimization.

Furthermore, safety comparisons should be interpreted cautiously because adverse-event definitions, reporting practices, and monitoring procedures varied across the included trials.

Additionally, limited data were available regarding biomarker subgroups (e.g., RAS and BRAF mutation status, microsatellite instability, and VEGF pathway markers) that may modulate treatment responses, and these were not amenable to subgroup analysis in the current work.

Additionally, the TAS-102 plus bevacizumab group included fewer patients than the other treatment groups, which may have influenced the precision of comparative estimates.

Finally, the placebo arms were pooled from multiple trials to serve as a common reference group, which may introduce confounding due to between-trial variation in patient selection and supportive care.

### 4.10. Future Directions

The findings of this study underscore several important directions for future research in the third-line management of mCRC. Most critically, prospective head-to-head randomized controlled trials directly comparing TAS-102 plus bevacizumab with fruquintinib are warranted, given that both agents target the anti-angiogenic pathway through complementary mechanisms and have demonstrated meaningful survival benefits in this setting. Such trials would provide definitive comparative evidence that is currently lacking and help establish a more evidence-based treatment hierarchy.

Beyond efficacy comparisons, future research should prioritize biomarker-driven patient selection strategies. The identification of predictive biomarkers, including RAS and BRAF mutation status, microsatellite instability, VEGF pathway markers, and tumor mutational burden, may allow for more precise matching of patients to the regimen most likely to confer benefit while minimizing unnecessary toxicity. In particular, patients with microsatellite instability-high (MSI-H) or deficient mismatch repair (dMMR) tumors may derive greater benefit from immune checkpoint inhibitor-based strategies than from cytotoxic or anti-angiogenic agents in the third-line setting, and this hypothesis merits dedicated prospective evaluation [34].

Additionally, the combination of regorafenib with fluorouracil (5-FU)-based chemotherapy at a reduced and optimized regorafenib dose of 80–120 mg represents a promising strategy that warrants prospective investigation. Dose-reduced regorafenib may preserve antitumor activity while substantially mitigating the toxicity burden that has historically limited its clinical use, and its combination with 5-FU could offer synergistic cytotoxic and anti-angiogenic effects in patients with refractory mCRC. Dedicated trials exploring this approach, particularly in patients who have not previously been exposed to regorafenib, are needed to define the optimal dosing schedule and patient population most likely to benefit.

Furthermore, the optimal sequencing of available third-line agents, particularly in relation to prior exposure to bevacizumab or anti-EGFR therapy in earlier lines, remains poorly defined and represents an important area for future investigation. Novel combination strategies, such as TKI plus immune checkpoint inhibitors or next-generation anti-angiogenic agents paired with chemotherapy backbones, should also be explored in biomarker-enriched cohorts. Collectively, these efforts will be essential in refining third-line treatment algorithms and ultimately improving outcomes for patients with refractory mCRC.

## 5. Conclusions

Our study demonstrated that all active regimens (TAS-102 monotherapy, TAS-102 plus bevacizumab, regorafenib, and fruquintinib) provided statistically significant and clinically meaningful improvements in OS and PFS compared with placebo. TAS-102 plus bevacizumab conferred the greatest survival benefit. Among the TKIs, fruquintinib showed numerically superior OS and significantly better PFS than regorafenib in indirect comparisons. Distinct toxicity profiles, primarily hematologic with TAS-102-based regimens and dermatologic and cardiovascular with TKIs, support individualized treatment selection. These findings suggest that TAS-102 plus bevacizumab demonstrated the most favorable efficacy profile among the evaluated regimens in this indirect comparison. However, these findings should be interpreted in light of the inherent limitations of reconstructed IPD and indirect cross-trial comparisons. Prospective head-to-head randomized trials are needed before definitive treatment recommendations or a treatment hierarchy can be established.

## Figures and Tables

**Table 1:** Characteristics of included studies.

Study Name	Year	Country	Intervention	Comparator	Reference
CORRECT Trial	2012	USA	Regorafenib	Placebo	[16]
SUNLIGHT Trial	2023	USA, Austria, France, Italy, Belgium, Spain, Brazil, Poland, Russia, Hungary, Denmark, Ukraine, Germany	Trifluridine/Tipiracil + Bevacizumab	Trifluridine/Tipiracil	[15]
FRESCO Trial	2018	China	Fruquintinib	Placebo	[17]
FRESCO-2 Trial	2023	USA, Italy, Spain, Germany, Australia, France, Belgium, Japan, Korea, Estonia	Fruquintinib	Placebo	[18]
RECOURSE Trial	2015	USA, Japan, Europe, Australia	TAS-102	Placebo	[14]

**Table 2:** Cox proportional hazards table for OS and PFS of all included groups.

	OS (HR)				PFS (HR)			
Characteristic	N	HR	95% CI	*p*-Value	N	HR	95% CI	*p*-Value
Treatment	3276				2892			
Placebo		—	—			—	—	
Fruquintinib		0.67	0.58, 0.76	<0.001		0.32	0.28, 0.36	<0.001
Regorafenib		0.68	0.57, 0.81	<0.001		0.39	0.32, 0.46	<0.001
TAS-102		0.70	0.61, 0.81	<0.001		0.41	0.36, 0.47	<0.001
TAS-102 + Bev		0.43	0.34, 0.55	<0.001		0.20	0.16, 0.24	<0.001

Abbreviations: CI = confidence interval, HR = hazard ratio.

**Table 3:** Total and specific grade 3/4 adverse events for regorafenib, TAS-102 plus bevacizumab, fruquintinib, and TAS-102.

G3/4 AEs	Regorafenib	TAS-102 + Bev	TAS-102	Fruquintinib	*p*-Value
**Total**	53.7	72.4	69.4	34	<0.001
**1.** **General**					
**Fatigue**	6.1	2.4	3.7	2.6	0.444
**Hand–foot skin reaction**	16.4	NA	NA	8.2	0.003
**2.** **Gastrointestinal**					
**Diarrhea**	2.9	0.8	2.8	3.1	0.329
**Nausea**	0.4	1.6	1.8	0.2	0.0533
**Vomiting**	0.6	0.8	1.9	0.7	0.109
**Bilirubin**	3.4	NA	8.6	0.6	<0.001
**ALT increase**	6.6	NA	1.9	1	<0.001
**AST increase**	5.9	NA	4.4	0.4	<0.001
**3.** **Cardiovascular**					
**Hypertension**	8	5.7	1.2	15.5	<0.001
**4.** **Hematological**					
**Platelets decrease**	2.7	2.8	2.8	2.5	0.995
**Anemia**	2.5	6.1	14.7	NA	<0.001
**Neutropenia**	2.2	43.1	19.2	NA	<0.001

## Data Availability

The data supporting the findings of this meta-analysis titled “A Meta-Analysis Comparing the Effectiveness and Safety of Different Third-line Management Options for Patients with Metastatic Colorectal Cancer” are available upon request from the corresponding author, Maen Abdelrahim.

## Supplementary Materials

The following supporting information can be downloaded at: https://www.mdpi.com/article/10.3390/cancers18142204/s1, File S1. The pooled reconstructed hazard ratio per study were compared with the published hazard ratios from the original trials; Table S1. G3/4 Adverse events including the placebo arm; Figure S1. Risk of Bias; Figure S2. Reconstructed Kaplan–Meier curve of progression-free survival (PFS) for the SUNLIGHT trial, comparing trifluridine/tipiracil (TAS-102) monotherapy against TAS-102 plus bevacizumab; Figure S3. Reconstructed Kaplan–Meier curve of progression-free survival (PFS) for the RECOURSE trial, comparing pooled placebo against trifluridine/tipiracil (TAS-102) monotherapy; Figure S4. Reconstructed Kaplan–Meier curve of progression-free survival (PFS) for the FRESCO-2 trial, comparing pooled placebo against fruquintinib monotherapy; Figure S5. Reconstructed Kaplan–Meier curve of progression-free survival (PFS) for the FRESCO-1 trial, comparing pooled placebo against fruquintinib monotherapy; Figure S6. Reconstructed Kaplan–Meier curve of progression-free survival (PFS) for the CORRECT trial, comparing pooled placebo against regorafenib monotherapy; Figure S7. Reconstructed Kaplan–Meier curve of overall survival (OS) for the SUNLIGHT trial, comparing trifluridine/tipiracil (TAS-102) monotherapy against TAS-102 plus bevacizumab; Figure S8. Reconstructed Kaplan–Meier curve of overall survival (OS) for the RECOURSE trial, comparing pooled placebo against trifluridine/tipiracil (TAS-102) monotherapy; Figure S9. Reconstructed Kaplan–Meier curve of overall survival (OS) for the FRESCO-2 trial, comparing pooled placebo against fruquintinib monotherapy; Figure S10. Reconstructed Kaplan–Meier curve of overall survival (OS) for the FRESCO-1 trial, comparing pooled placebo against fruquintinib monotherapy; Figure S11. Reconstructed Kaplan–Meier curve of overall survival (OS) for the CORRECT trial, comparing pooled placebo against regorafenib monotherapy.
