# Supplementary material for: Comparing the Effectiveness and Safety of Different Third-Line Management Options for Patients with Metastatic Colorectal Cancer: A Systematic Review and Reconstructed Individual Patient Data Meta-Analysis
Source: Cancers (Basel). 2026 Jul 8;18(14):2204. doi: 10.3390/cancers18142204 (PMC13407048; doi:10.3390/cancers18142204)
Supplement: Supplementary file 1 [file cancers-18-02204-s001.zip › cancers-4394338-supplementary.pdf]

Table S1. G3/4 adverse events including the placebo arm.

G3/4 AE	Regorafenib	Placebo	TAS-102 + Bev	TAS-102	Fruquintinib	p_value
**Total**	53.7	18.1	72.4	69.4	34	p < 0.001
**Fatigue**	6.1	3	2.4	3.7	2.6	0.526
**Diarrhea**	2.9	0.4	0.8	2.8	3.1	0.00182
**Hypertension**	8	1.4	5.7	1.2	15.5	p < 0.001
**Decr. Platelets**	2.7	0.4	2.8	2.8	2.5	0.0426
**Abdominal**	0.3	0.2	2	2.2	NA	0.0525
**Nausea**	0.4	0.5	1.6	1.8	0.2	0.0424
**Vomiting**	0.6	0.3	0.8	1.9	0.7	0.0283
**Anemia**	2.5	0.2	6.1	14.7	NA	p < 0.001
**Bilirubin**	3.4	1.8	NA	8.6	0.6	p < 0.001
**Hand–Foot**	16.4	0.1	NA	0	8.2	p < 0.001
**Dysphonia**	0.3	0	NA	NA	0	1
**ALT**	6.6	0.7	NA	1.9	1	p < 0.001
**AST**	5.9	1.2	NA	4.4	0.4	p < 0.001
**Neutropenia**	2.2	0	43.1	19.2	NA	p < 0.001

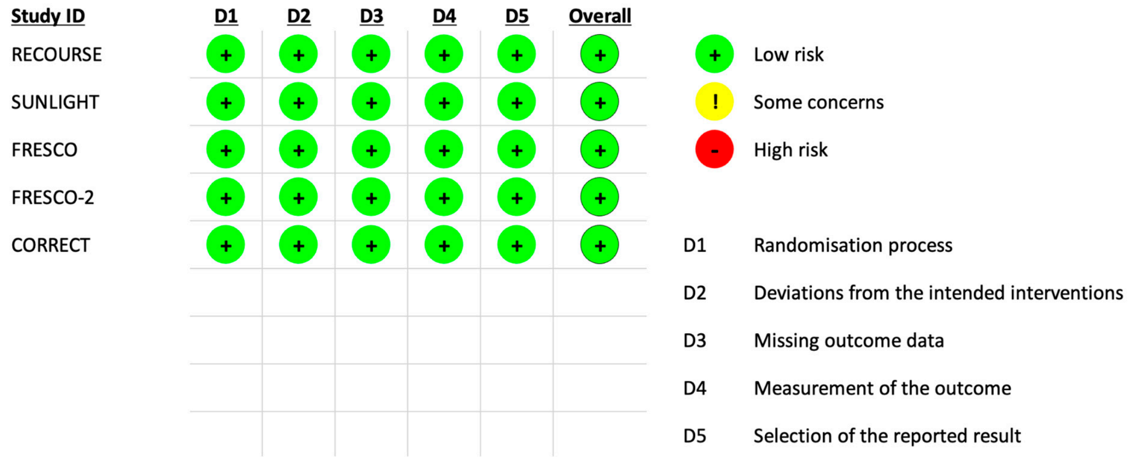

Figure S1. Risk of bias.

survival (PFS)

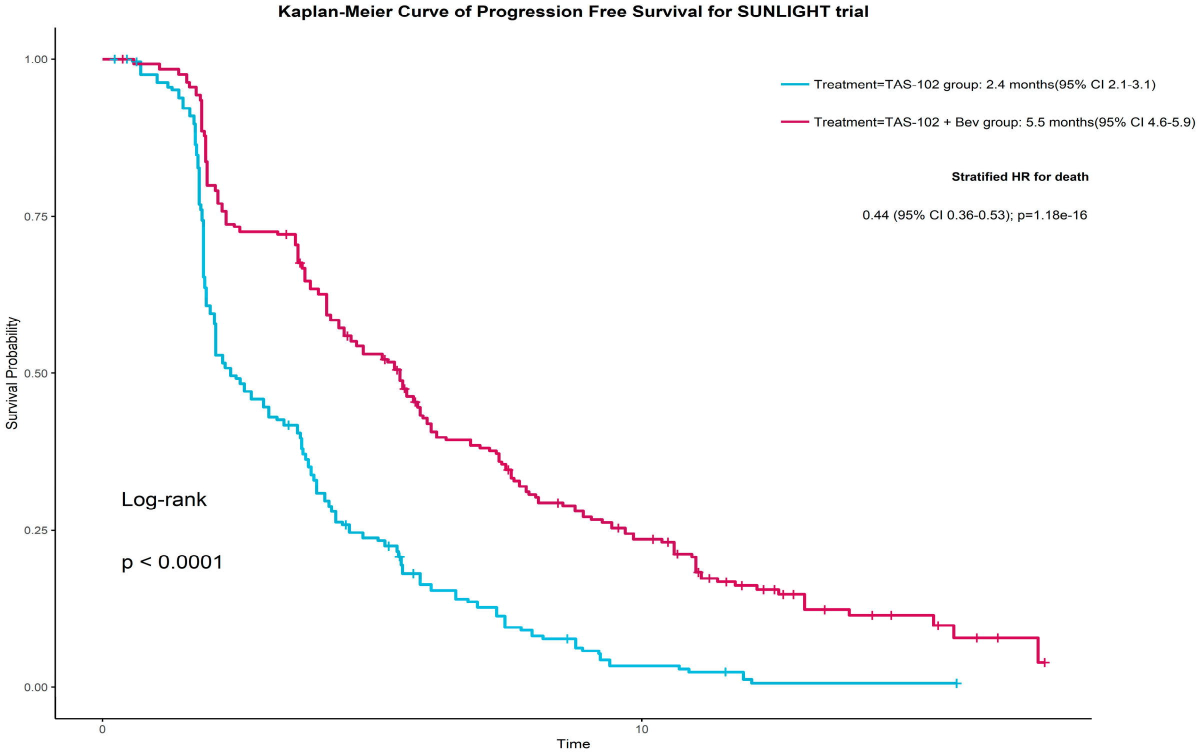

Figure S2. Reconstructed Kaplan–Meier curve of progression-free survival (PFS) for the SUNLIGHT trial, comparing trifluridine/tipiracil (TAS-102) monotherapy against TAS-102 plus bevacizumab.

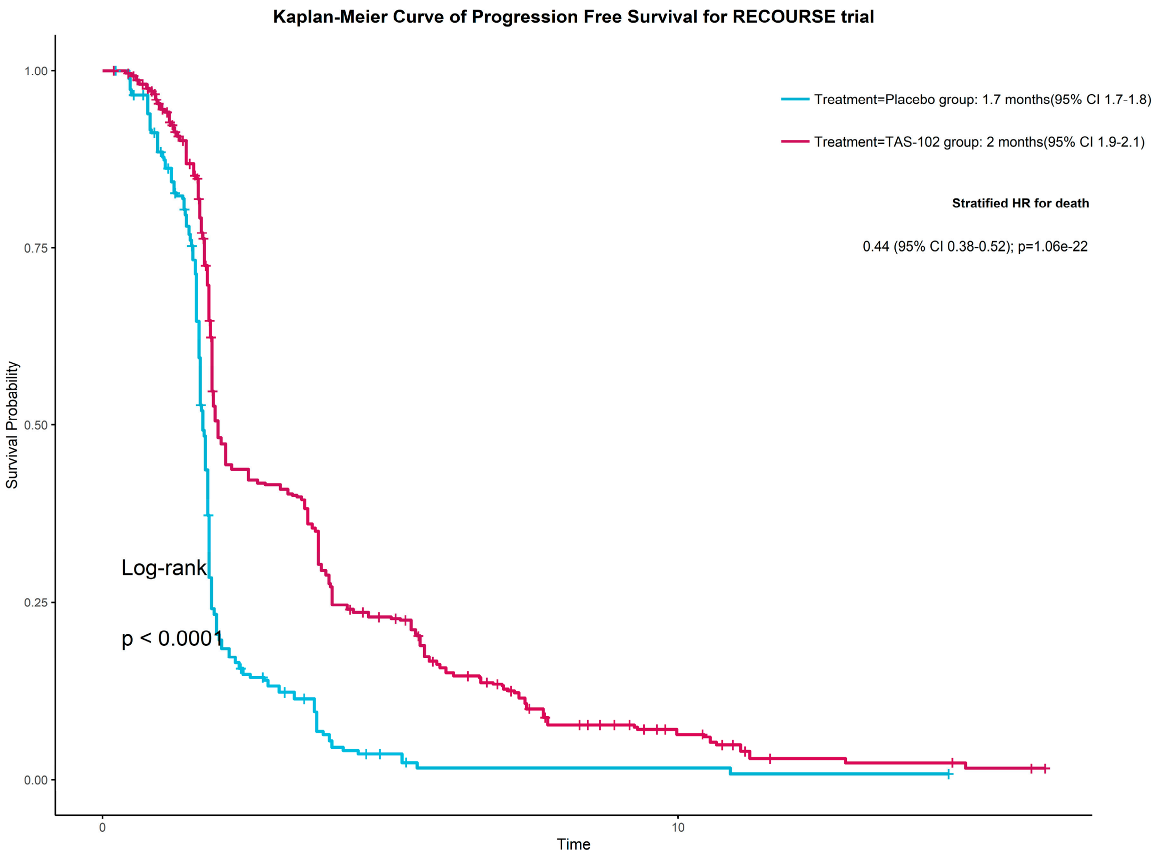

Figure S3. Reconstructed Kaplan–Meier curve of progression-free survival (PFS) for the RECOURSE trial, comparing pooled placebo against trifluridine/tipiracil (TAS-102) monotherapy.

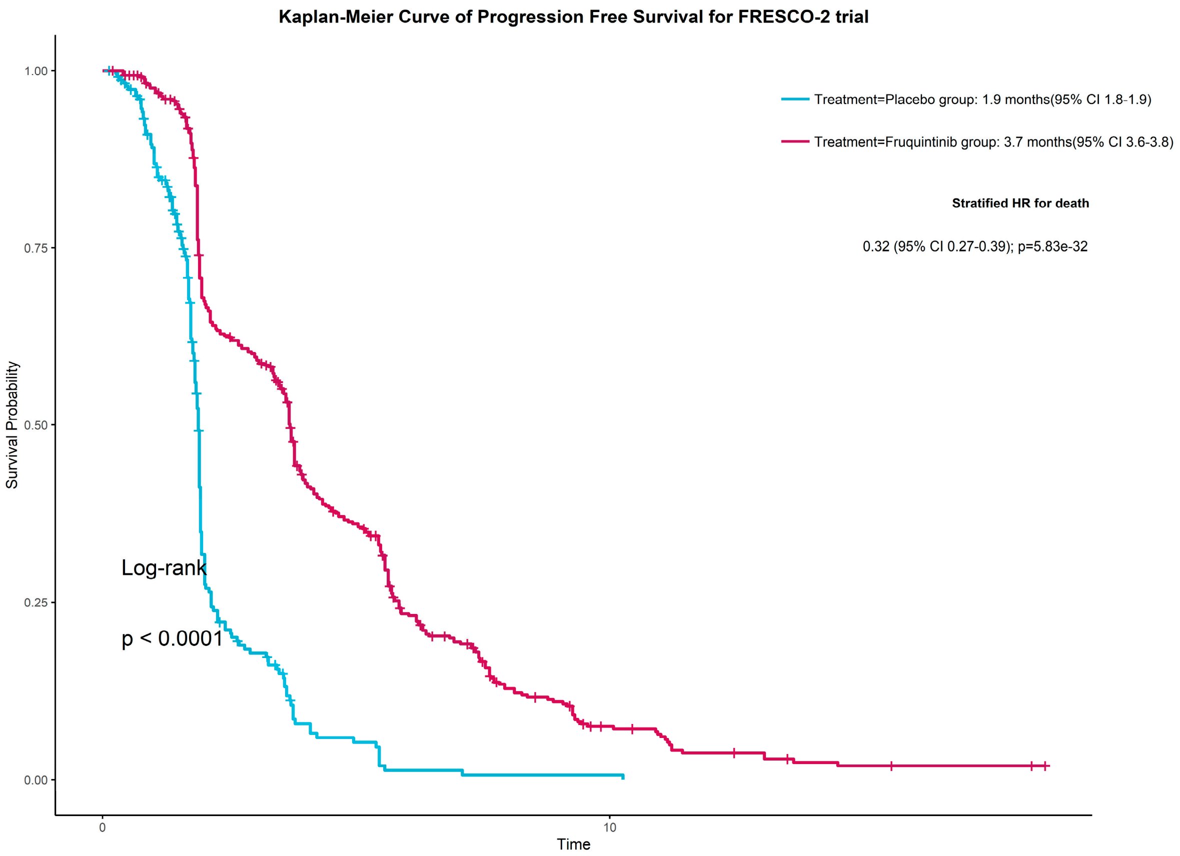

Figure S4. Reconstructed Kaplan–Meier curve of progression-free survival (PFS) for the FRESCO-2 trial, comparing pooled placebo against fruquintinib monotherapy.

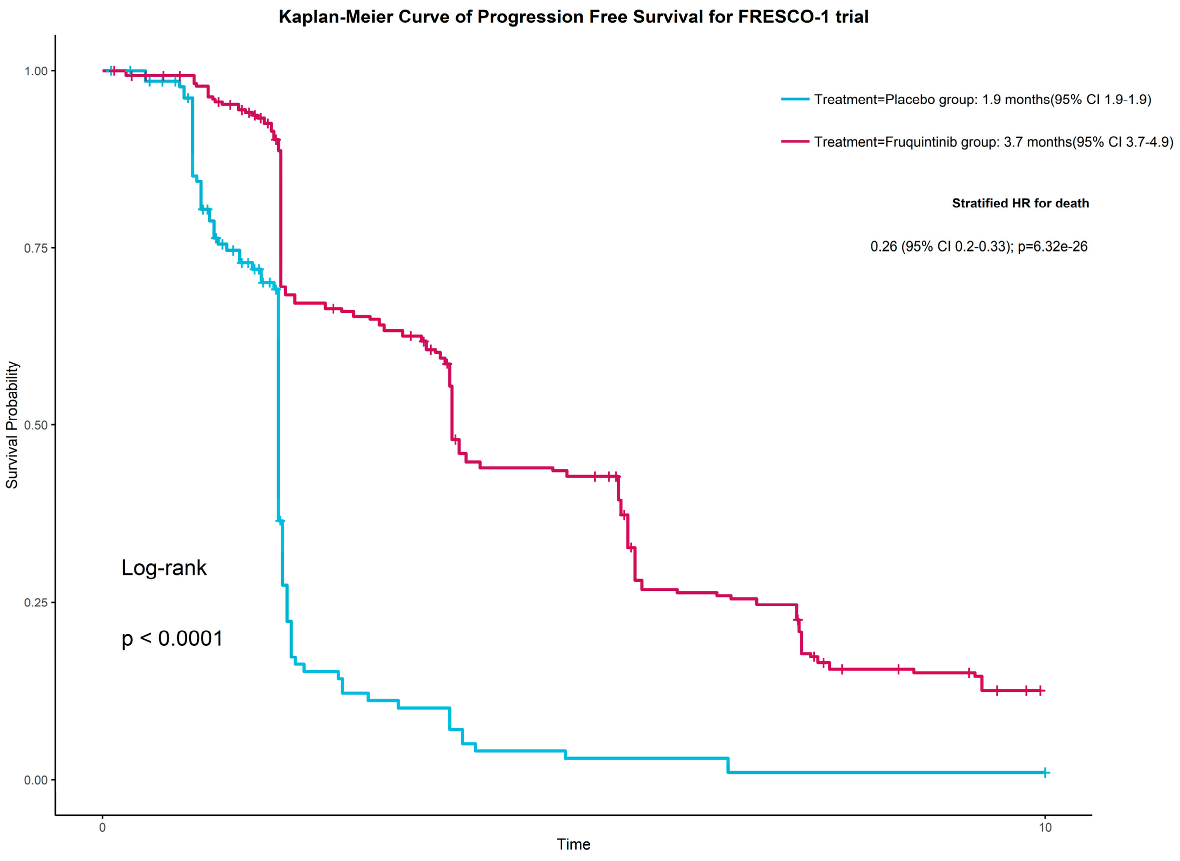

Figure S5. Reconstructed Kaplan–Meier curve of progression-free survival (PFS) for the FRESCO-1 trial, comparing pooled placebo against fruquintinib monotherapy.

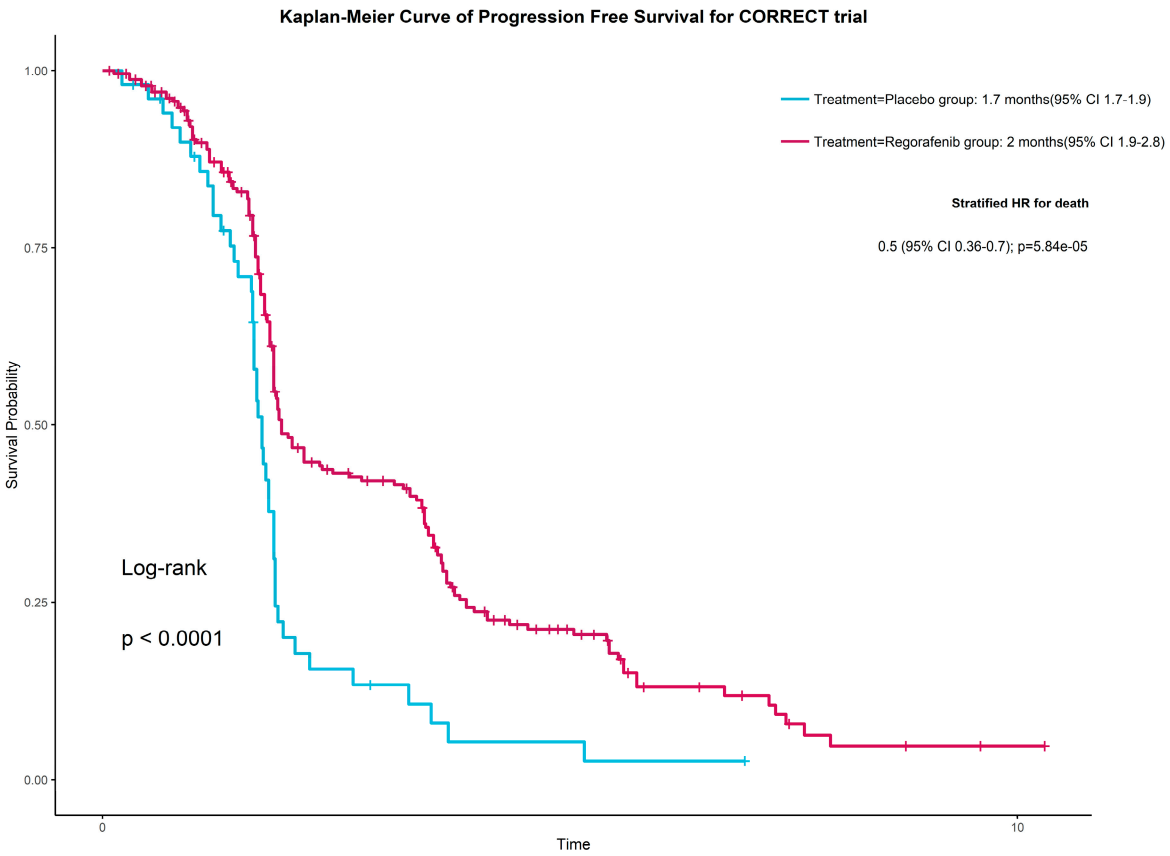

Figure S6. Reconstructed Kaplan–Meier curve of progression-free survival (PFS) for the CORRECT trial, comparing pooled placebo against regorafenib monotherapy.

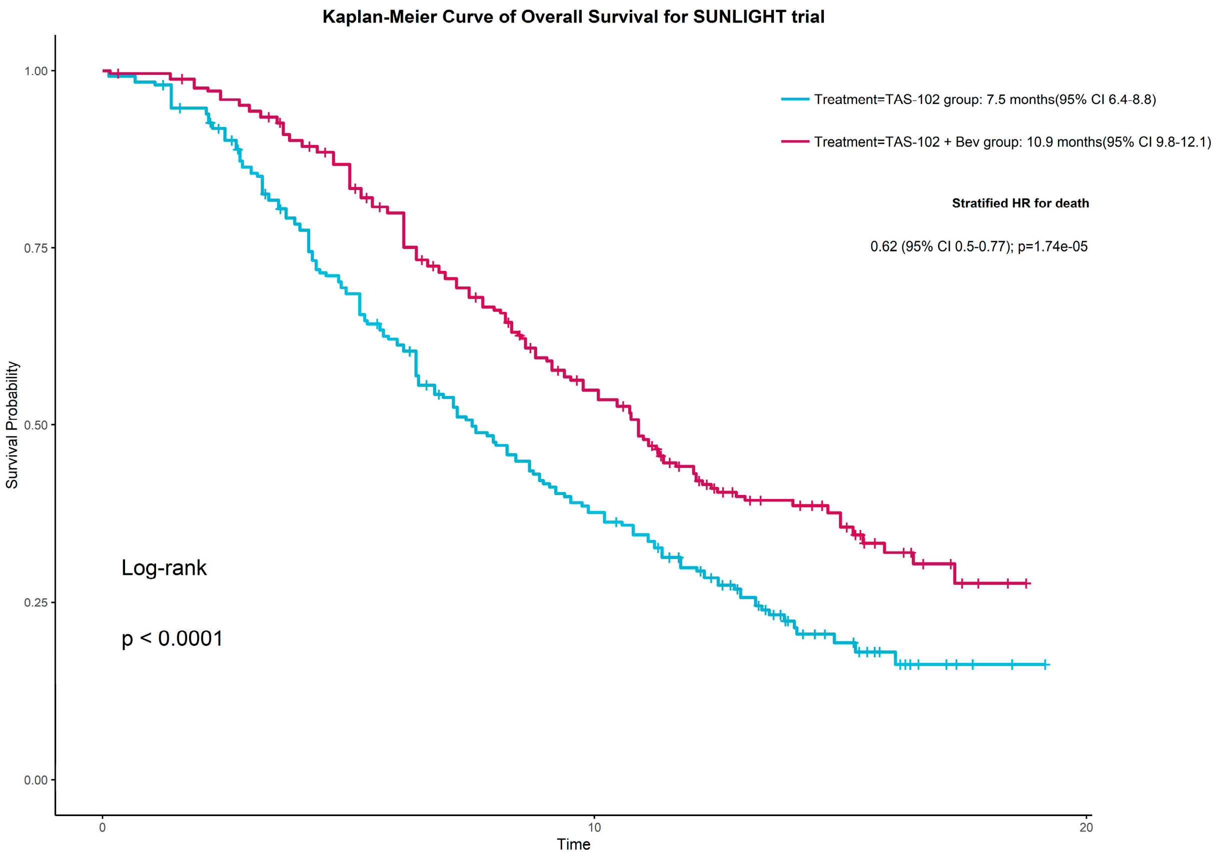

Figure S7. Reconstructed Kaplan–Meier curve of overall survival (OS) for the SUNLIGHT trial, comparing trifluridine/tipiracil (TAS-102) monotherapy against TAS-102 plus bevacizumab.

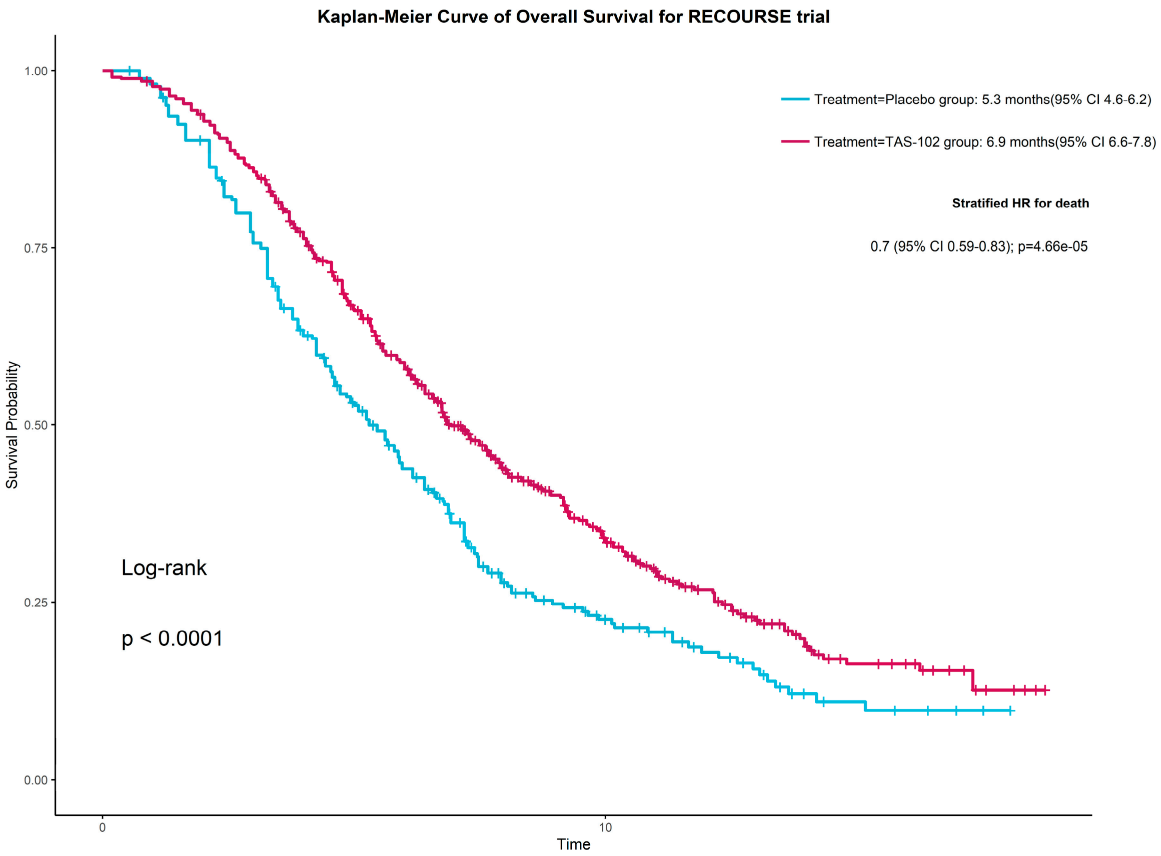

Figure S8. Reconstructed Kaplan–Meier curve of overall survival (OS) for the RECOURSE trial, comparing pooled placebo against trifluridine/tipiracil (TAS-102) monotherapy.

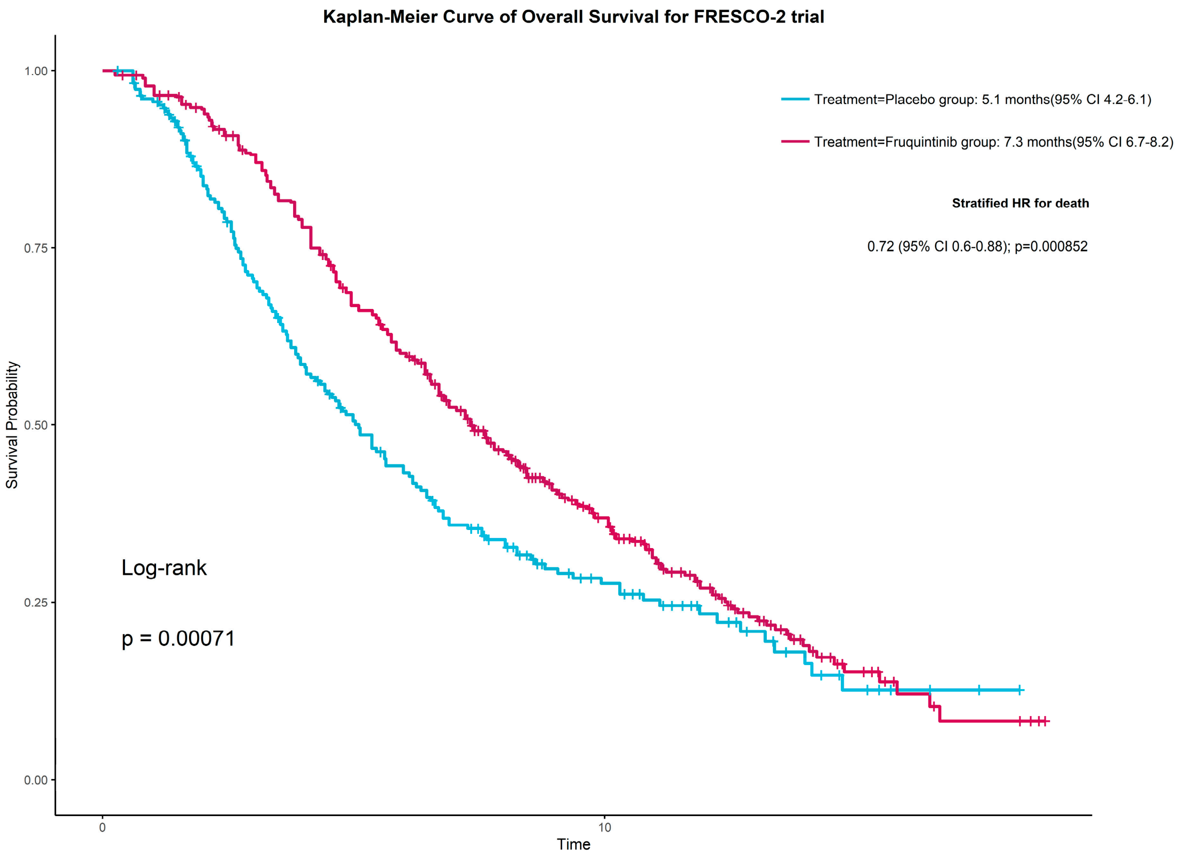

Figure S9. Reconstructed Kaplan–Meier curve of overall survival (OS) for the FRESCO-2 trial, comparing pooled placebo against fruquintinib monotherapy.

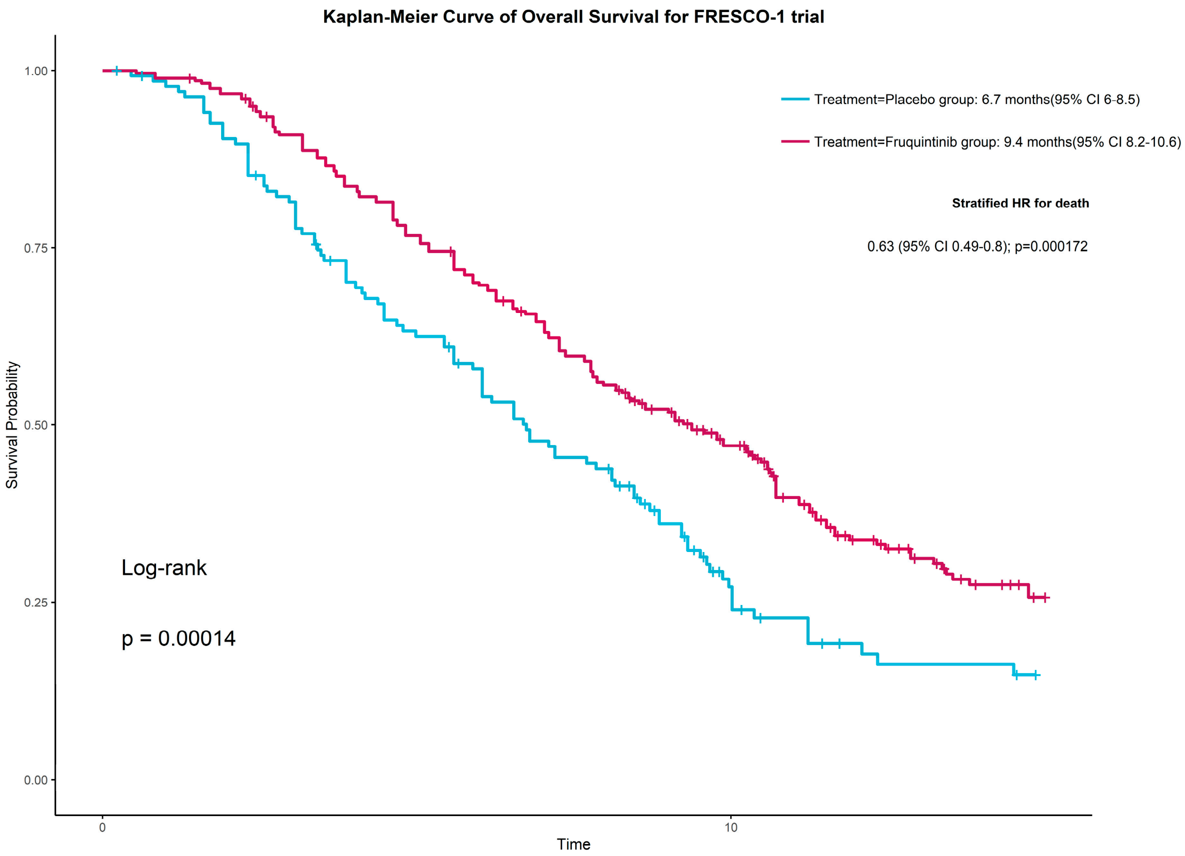

Figure S10. Reconstructed Kaplan–Meier curve of overall survival (OS) for the FRESCO-1 trial, comparing pooled placebo against fruquintinib monotherapy.

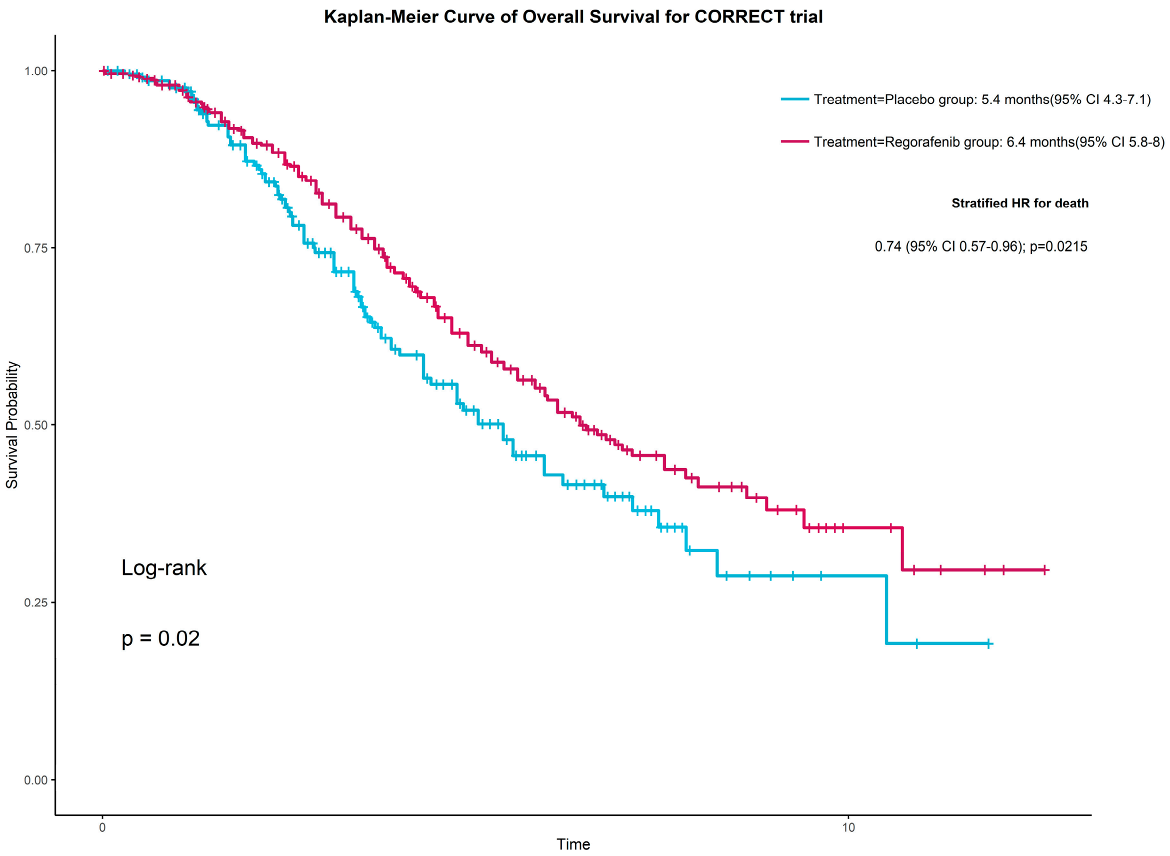

Figure S11. Reconstructed Kaplan–Meier curve of overall survival (OS) for the CORRECT trial, comparing pooled placebo against regorafenib monotherapy.
